# The MVA-VP2-NS1-2A-NS2-Nt vaccine candidate provides heterologous protection in sheep against bluetongue virus

**DOI:** 10.3389/fimmu.2025.1566225

**Published:** 2025-05-05

**Authors:** Luis Jiménez-Cabello, Sergio Utrilla-Trigo, Miguel Illescas-Amo, Karen Rodríguez-Sabando, Julio Benavides-Silván, Eva Calvo-Pinilla, Javier Ortego

**Affiliations:** ^1^ Centro de Investigación en Sanidad Animal (CISA), Instituto Nacional de Investigación y Tecnología Agraria y Alimentaria (INIA), Valdeolmos, Madrid, Spain; ^2^ Universidad Autónoma de Madrid (UAM), Escuela de doctorado, Madrid, Spain; ^3^ Instituto de Ganadería de Montaña (CSIC-Universidad de León), León, Spain

**Keywords:** bluetongue virus (BTV), orbivirus, vaccine, MVA, DIVA, multiserotype, sheep

## Abstract

Bluetongue (BT) is an important arthropod-borne livestock disease transmitted by *Culicoides* midges. The etiological agent, Bluetongue virus (BTV), can lead to severe economic losses due to reduced productivity and trade restrictions. Nowadays, classical vaccines based on inactivated viruses are used to control outbreaks but do not confer multiserotype protection, which reinforces the idea of pursuing research into developing strategies that enhance the immune response directed to conserved antigenic regions, aiming broader protection across multiple serotypes. Recently, we described a vaccine candidate that confers full protection against a homologous serotype of BTV based on recombinant Modified Vaccinia Virus Ankara (MVA) co-expressing the highly conserved BTV nonstructural protein NS1 and the N-terminal end of NS2 along with protein VP2 of BTV-4. In this work, we evaluated the multiserotype protective capacity of this recombinant vaccine candidate in sheep after infection with the heterologous virus BTV-8, achieving a significant blockade of viral replication and attenuation of the clinical signs induced by BTV. After infection, vaccinated animals showed more regulated pro-inflammatory cytokine levels compared to non-vaccinated sheep. In addition, we noticed the induction of potent T cell immune responses specific to NS1 and NS2-Nt proteins of BTV, mainly based on CD8+ T cells, which could mediate the protection against BTV-8. Moreover, stimulated immunized sheep PBMCs with BTV antigens triggered the secretion of IL-6, IL-1β, IL-1α, IL-17a, IL-10 and IFN-γ, cytokines that play crucial roles in initiating immune responses.

## 1 Introduction

Bluetongue is an arthropod-borne disease of ruminants transmitted during blood-feeding by some species of biting midges of the genus *Culicoides* (Diptera: *Ceratopogonidae*) (1). This WOAH-listed disease, caused by Bluetongue virus (BTV), affects predominantly sheep and cattle, but other domestic and wild ruminants are sensitive to infection and disease (2). To date, more than 29 serotypes of BTV have been identified, being recognized by cross-neutralization and confirmed by phylogenetic analysis of segment 2 encoding for VP2 protein (3). BTV is worldwide distributed and new incursions into non-endemic areas are frequent. Indeed, new introductions of the virus occurs in Europe each year since 1998, involving eleven distinct BTV serotypes (BTV-1, 2, 4, 6, 8, 9, 11, 14, 16, 25, and 27) (4). Recently, a novel BTV serotype 3 strain (BTV-3 NET2023) emerged in northern Europe for the first time in 2023 from an unknown origin (5). BTV has been estimated to cause direct and indirect losses of over $3 billion per year worldwide (6, 7). Vaccination strategies against this disease are based on conventional approaches, such as inactivated or live attenuated vaccines (LAVs) (8, 9). Despite their crucial role in BTV control, these vaccines are serotype-specific. The co-circulation of multiple and new serotypes is becoming more frequent in many regions, so the development of an effective multiserotype vaccine remains an important goal for the safe and cost-effective control of this disease.

BTV belongs to the genus *Orbivirus* within the family *Sedoreoviridae* (10). BTV is a non-enveloped virus composed of an icosahedral capsid inside of which ten double-stranded RNA (dsRNA) segments are contained (11, 12). The BTV genome encodes for seven structural proteins (VP1-VP7) and five/six nonstructural proteins (NS1, NS2, NS3/3A, NS4 and NS5) (11–15). Among them, protein VP2 is the main inductor of neutralizing antibodies (NAbs) although it is highly variable among BTV serotypes, showing higher sequence variation in specific regions exposed to antigenic selection pressure (3). As scarce cross-neutralizing relationships exist between BTV serotypes (16), other BTV antigens highly conserved among serotypes should be contemplated for achieving multiserotype protection. In this sense, two of the most conserved BTV proteins among serotypes, NS1 and NS2 (or its N-terminal half) elicit cross-reactive cellular immune responses. However, despite that these antigens provide partial protective immunity against homologous BTV serotypes in natural hosts of the disease (17–19), the heterologous protective potential has only been previously evaluated in IFNAR(-/-) mice.

Viral vector vaccines platforms are potential DIVA and safe vaccines, as the lack of infectious BTV is guaranteed (8). Different viruses have been explored as vectors for the development of vaccines expressing one or more BTV proteins intracellularly (20). The combination of both arms of the adaptive immune response, virus NAbs and cytotoxic T lymphocytes (CTLs), is crucial for the development of long lasting immunity against BTV (21, 22), which should guide BTV vaccine development. Following this rationale, we have recently developed a MVA-based vaccine candidate against BTV co-expressing the structural protein VP2 of BTV-4 along with nonstructural proteins NS1 and NS2-Nt, highly conserved among serotypes. This vaccine candidate showed high efficacy of protection against the homologous and virulent BTV-4 Morocco strain (BTV-4M) in sheep, blocking viral replication and the progression of disease after challenge (23). However, the multiserotype potential of this promising recombinant vaccine candidate has not been evaluated yet in a bluetongue virus natural host.

In this work, we have evaluated the multiserotype protective capacity of the MVA co-expressing VP2 along with the immunogenic NS1 and NS2-Nt proteins of BTV serotype 4 (BTV-4) against a heterologous BTV serotype 8 (BTV-8) infection in sheep. Besides, we characterized the cell-mediated protective immune response induced by this vaccine candidate in the BTV natural hosts.

## 2 Materials and methods

### 2.1 Cells lines and viruses

Chicken embryo fibroblasts (DF-1) (ATCC, Cat. No. CRL-12203) were grown in Dulbecco’s Modified Eagle’s medium (DMEM) (Biowest, Nuaillé, France) supplemented with 2mM glutamine, 1% penicillin/streptomycin (Gibco, Waltham, MA, USA) and 10% fetal calf serum (FCS) (Gibco, Waltham, MA, USA). Green monkey kidney cells (Vero) (ATCC, Cat. No. CCL-81) were grown in DMEM supplemented with 2mM glutamine, 1% penicillin/streptomycin and 5% FCS.

BTV serotype 4 Morocco strain (MOR2009/09) (BTV-4M) and BTV serotype 8 (BEL/2006) were used in the experiments. BTV-4M strain is a reassortant strain between BTV-1 (segments 1, 4, 5, 7, 9, 10) and BTV-4 (segments 2, 3, 6, 8) isolated from sheep blood in KC insect cells (24, 25). The MVA-VP2-NS1-2A-NS2-Nt, previously generated (23), was used for sheep immunization. Virus stocks and titrations were performed by standard methods previously described (26, 27).

### 2.2 Sheep

A total of 8 sheep (Spanish *Ovis aries* “Churra” breed) were used for the studies. All sheep used were matched for age (4 months). Sheep were housed under pathogen-free conditions and allowed to acclimatize to the biosafety level 3 (BSL3) animal facilities at the Animal Health Research Center (CISA-INIA, CSIC), Madrid, before use.

### 2.3 Sheep immunization and challenge

All sheep involved in the experiment were negative to BTV by ELISA. One group of sheep (n=4) was intramuscularly immunized following a homologous primer-boost strategy consisting of two doses of 10^8^ PFU of MVA-VP2-NS1-2A-NS2-Nt administered four weeks apart. A group of sheep was left untreated (control). Pre-challenge blood samples were collected from all animals. Non-immunized and immunized sheep were subcutaneously challenged with a dose of 10^5^ PFU of BTV-8 at three weeks post-boost. After virus challenge, blood collection for virological analyses was conducted by specialized veterinary personal at 0, 3, 5, 7, 10, 12, 14, and 18 d.p.i. Rectal temperatures measurements were conducted every day from 4 days prior to challenge until 18 d.p.i. At day 18 post-infection all sheep were euthanized.

### 2.4 Viraemia and RNAemia analysis by plaque assay and RT-qPCR, respectively

Blood samples were collected at 0, 3, 5, 7, 10, 12, 14, and 18 d.p.i. from sheep with EDTA as anti-coagulant. RNA was extracted from 50 µL of blood using TRIzol Reagent (Sigma Aldrich, St. Louis, MO, USA) following the protocol established by the manufacturer. RNAemia was analyzed in duplicate by real-time RT-qPCR specific for BTV segment 5 (encoding NS1). The real-time RT-qPCR specific for BTV segment 5 was performed using primers and probe described by Toussaint et al. (28). Only Ct values lower than 38 were considered indicative of RNAemia (positive), according to the cut-off established by Toussaint et al. (28). “No Ct” values were considered as a Ct of 45, last cycle of the RT-qPCR.

For the analysis of viraemia by plaque assay, 50 µL of sheep blood were washed with 450 µL of PBS and centrifuged at 3000 rpm for 10 minutes. Thereafter, supernatant was removed, and pellet was lysed in 450 µL of sterile water for 2 minutes. Cell lysis was stopped by adding 50 µL of PBS10X. Then, samples were inoculated into 12-well plates containing semi-confluent monolayers of Vero cells. Following incubation for 1 h, an agar overlay (DMEM-10%-FBS-0.4%-Noble Agar, Becton Dickinson, MD, USA) was added and plates were incubated for 5 days at 37°C in 5% CO_2_. Plaques were fixed with 10% formaldehyde and visualized with 2% crystal violet.

### 2.5 Blood measurements

A multiparameter autohematology analyzer (BC-5300 Vet; Mindray, China) was used to determine the total and differential cell counts in sheep blood for each group collected into EDTA tubes.

### 2.6 Determination of circulating levels of cytokines

Sera from immunized and non-immunized sheep were collected the day before the challenge and at 3 and 5 d.p.i. Circulating cytokine levels were analyzed using a multiplex fluorescent bead immunoassay for quantitative detection of sheep cytokines (Millipore’s MILLIPLEX Mouse Cytokine kit, Burlington, MA, USA). Samples were analyzed for IL1-α, IL-1β, IL-4, IL-6, IL-10, IFN-γ and TNF, with a MAGPIX system (Luminex Corporation, Austin, TX, USA). Values of blank samples were subtracted from values of samples.

### 2.7 ELISpot

ELISpot plates with Immobilon-P membranes (Millipore) were first permeabilized using 35% ethanol in sterile water, then washed twice with PBS. They were coated with 50 μg of the capture antibody, bovine anti-IFN-γ (MT17.1, Mabtech) showing sheep reactivity, at a concentration of 2.5 μg/ml in PBS, and incubated overnight at 4°C. Afterward, the plates were blocked with complete medium for 1 hour at 37°C. PMBCs from sheep at three weeks after boost were seeded at 2x10^5^ cells per well, then stimulated with a pool of 5 μg/ml NS1 peptides, 5 μg/ml of the NS2-Nt protein, Phorbol 12-myristate 13-acetate phytohemagglutinin (PHA) (Sigma) as a nonspecific stimulus (4 μg/ml), or an irrelevant peptide (protein Gn of Rift Valley Fever virus, RVFV) as the negative control, for 18h or left untreated in RPMI 1640 medium supplemented with 10% FCS. Following the 18-hour incubation, the plates were washed with distilled water to lyse the cells, followed by five washes with PBS. Next, 50 µL per well of biotin-conjugated anti-IFN-γ antibody (MT307, Mabtech), diluted 1:100 in PBS, was added and incubated at room temperature for 2 hours in the dark. This was followed by the addition of 50 µL of streptavidin-HRP conjugate (BD Biosciences), diluted 1:1,000 in PBS, and incubated for 1 hour. Finally, 50 µL of TMB developing solution (Mabtech) was added to each well until spots became visible, followed by a wash with water. After drying the membranes, the number of spots in each well was determined using an AID iSpot Reader System (Autoimmun Diagnostika, Strassberg, Germany).

### 2.8 *Ex-vivo* flow cytometric analysis

To evaluate the immunogenicity of the MVA-VP2-NS1-2A-NS2-Nt in sheep, blood samples of immunized and non-immunized sheep were harvested for peripheral blood mononuclear cell (PBMC) isolation and subsequent analysis by intracellular cytokine staining assays (ICS). Fresh PBMC were isolated from whole blood using Ficoll-Hypaque density gradient centrifugation.

A total of 10^6^ PMBCs per well were stimulated with 5 μg/ml of a pool of NS1 peptides (**Table 1**), 5 μg/ml of the NS2-Nt protein, concanavalin A (ConA) as a non-specific stimulus (4 μg/ml) for 18h or left untreated in RPMI 1640 medium supplemented with 10% FCS. Six hours before the assay, brefeldin A (5 µg/ml) was added. After stimulation, cells were washed with PBS-1%-FBS, stained for the surface markers, fixed with PBS-1%-FBS-1%-Saponine-4%-PFA, permeabilized with PBS-1%-FBS-1%-Saponine, and stained intracellularly using the fluorochrome conjugated antibodies anti-bovine IFN-γ mAb (MT307, Mabtech), conjugated to PF647P, and anti-bovine IL-2 mAb (MT3B3, Mabtech), conjugated to PF647P. Fluorochrome conjugated antibodies mouse anti-Sheep CD4-FITC, CD8-PE and Workshop Cluster 1 (WC1)-FITC (MCA2213F, MCA2216PE, MCA2222F, Biorad) were used for the analysis of extracellular receptor molecules. Data were acquired for FACS analysis on a FACS Celesta Sorp platform (Becton Dickinson, Franklin Lakes, NJ, USA). Analyses of the data were performed using FlowJo software version x10.9 (Tree Star, Ashland, OR, USA). The number of lymphocyte-gated events was 5x10^5^. Lymphocytes were initially gated on the basis of their forward and side scatter properties. Then, CD4+, CD8+ or WC1+ (gamma delta, γδ) lymphocytes expressing IFN-γ or IL-2 were selected for the analysis. Gating strategies used to identify T-cell populations are shown in **Supplementary Figure S1**.

**Table 1:** Peptides selected from BTV NS1 protein using the epitope prediction in H-2 Db haplotype.

Position	Sequence	IEDB	SYFPEITHI	BYMAS
125	SALVNSERV	0.2	28	51.480
152	GQIVNPTFI	0.2	28	720.000
14	YANATRTFL	0.7	16	2.600
222	IQLINFLRM	0.2	25	792.000

A combination of three epitope T prediction algorithms (IEDB, SYFPEITHI and BYMAS) was used to select peptides from NS1 proteins of BTV-4.

### 2.8 Plaque reduction neutralization test

Two-fold dilutions (from 1:5) of heat inactivated sheep sera (56°C for 30 minutes) were incubated with 100 PFU of BTV-4M or BTV-8, for 1h at 37°C. Then, samples were inoculated into 12-well plates containing semi-confluent monolayers of Vero cells. Following incubation for 1h, an agar overlay (DMEM-10%-FBS-0.4%-Noble Agar (Becton Dickinson, MD, USA)) was added and plates were incubated for 5 days at 37°C in 5% CO_2_. Plaques were fixed with 10% formaldehyde and visualized with 2% crystal violet-PBS. PRNT50 titer was calculated as the highest dilution of serum that neutralized 50% of the control virus input.

### 2.9 Statistical analysis

Data were analyzed using GraphPad Prism version 8.0.1 (GraphPad Software, San Diego, CA, USA). Comparisons of mean responses between groups for the ICS, cytokine levels, PRNT50 and ELISA assays as well as data on rectal temperature, viraemia, RNAemia and hematological values were conducted by two-way ANOVA with a *post hoc* Tukey test for multiple comparisons. A p-value lower than 0.05 was considered significant in all cases.

### 2.10 Ethics statement

Animal experimental protocols were approved by the Ethical Review Committee at the INIA-CISA and Comunidad de Madrid (Permit number: PROEX 060.7/21) in strict accordance with EU guidelines 2010/63/UE about protection of animals used for experimentation, and other scientific purposes and Spanish Animal Welfare Act 32/2007.

## 3 Results

### 3.1 Protection conferred by MVA-VP2-NS1-2A-NS2-Nt in sheep against BTV-8

**Figure 1:**
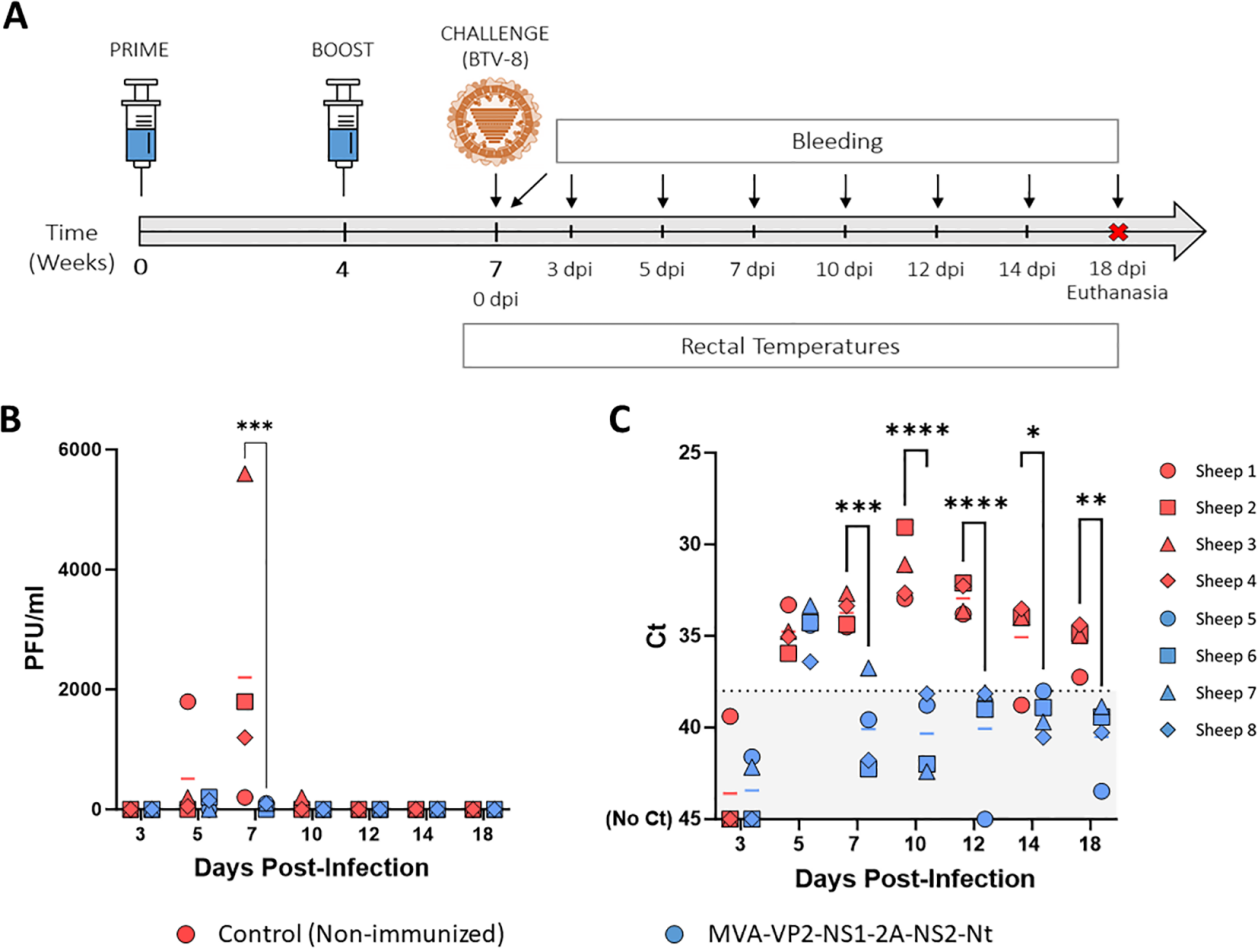
Protection of immunized sheep against a heterologous virulent challenge with BTV-8. **(A)** A group of sheep (n=4) was immunized following a homologous prime-boost regimen consisting of two doses of MVA-VP2-NS1-2A-NS2-Nt. A second group was left untreated (Control). Immunized and non-immunized sheep were challenged with BTV-8. **(B)** Titers of BTV-8 recovered in blood of sheep after viral inoculation. Points represent individual PFU/ml values and lines represent mean Log PFU/ml of each group. **(C)** RNAemia analyzed by RT-qPCR of non-immunized and immunized sheep after viral challenge. Presence of virus in blood and expression of mRNA of segment 5 (encoding NS1 protein) was quantified. Results were expressed as Ct (left y-axis). The real-time RT-qPCR specific for BTV segment 5 was performed as described by Toussaint et al. (28). Cut-off Ct ≥ 38 (dotted black line). Points represent individual Ct values and lines represent mean Ct of each group. *P value  <  0.05, **P value  <  0.002, ***P value  <  0.001, ****P value  <  0.0001 using two-way ANOVA (*post hoc* Tukey test for multiple comparisons).

As stated above, the protective capability of the MVA-VP2-NS1-2A-NS2-Nt vaccine candidate against a homologous BTV-4 infection has been confirmed in sheep (23). To test the multiserotype protective capacity of the MVA co-expressing proteins VP2, NS1 and NS2-Nt of BTV-4, we immunized sheep following a prime-boost strategy consisting of two doses (1x10^8^ PFU) of MVA-VP2-NS1-2A-NS2-Nt, administered in a four-week interval. Three weeks after the booster, sheep were subcutaneously challenged with 10^5^ PFU of the heterologous BTV-8 (**Figure 1A**). Thereafter, rectal temperatures, viraemia, RNAemia and hematological parameters were measured at different days post-infection.

After challenge, three out of four non-immunized control sheep developed viraemia at 5 d.p.i. (mean virus titer = 512.5 PFU/ml) and all of them displayed high levels infectious virus in blood at 7 d.p.i. (mean virus titer = 2200 PFU/ml) (**Figure 1B**). Besides, one control sheep was still viremic at 10 d.p.i. Importantly, immunization with MVA-VP2-NS1-2A-NS2-Nt completely prevented two out of four sheep from developing viraemia at 5 d.p.i. and the mean virus titer of the group (87.5 PFU/ml), was considerably lower compared to the control sheep group. Moreover, the immunized sheep had very low levels of infectious virus in blood at day 7 post-infection (mean virus titer = 75 PFU/ml), being significantly (p-value < 0.001) lower to those of the control group (**Figure 1B**). Importantly, we did not detect infectious virus in any immunized sheep at 10 d.p.i. and onwards (**Figure 1B**). All non-immunized animals also showed detectable levels of viral RNA in blood from day 5 post-infection to the end of the experiment, with peak levels between 7 and 12 d.p.i. (**Figure 1C**). In contrast, the MVA-VP2-NS1-2A-NS2-Nt just displayed the RNAemic status at 5 d.p.i. but at the remaining days post-infection they displayed undetectable (Ct ≥ 38) or nearly undetectable levels of RNA (**Figure 1C**).

**Figure 2:**
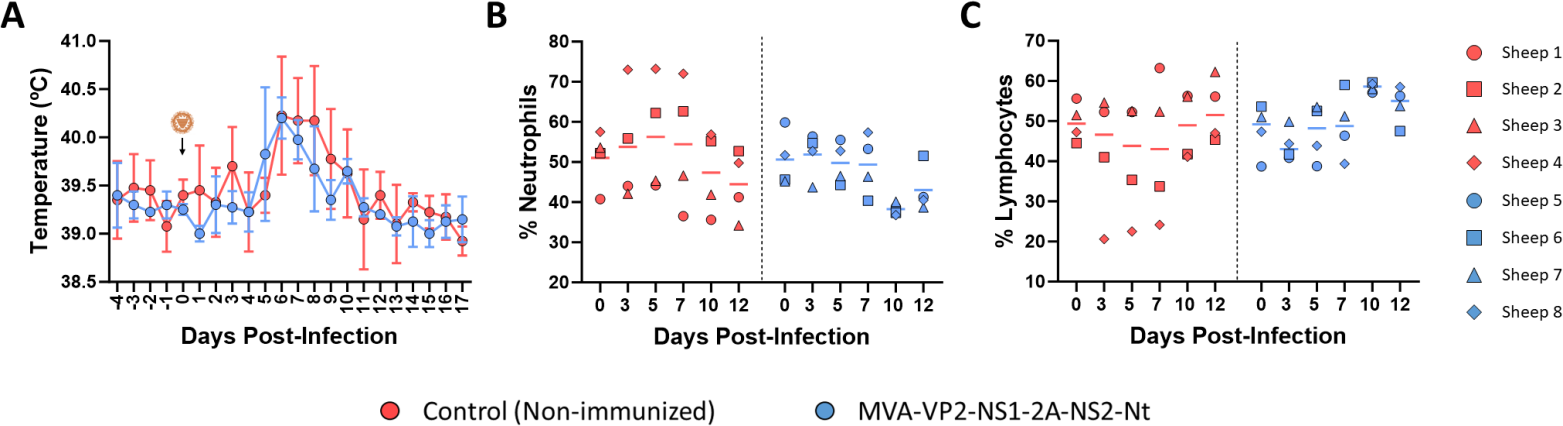
Changes in rectal temperatures and hematologic parameters in immunized and non-immunized sheep after BTV-8 challenge. **(A)** Rectal temperatures recorded before and after challenge. The day of challenge (0 d.p.i.) is indicated. Points represent mean rectal temperature value for each group and error bars represent SD. **(B, C)** Percentages of lymphocytes and neutrophils in blood from immunized sheep after challenge with BTV-8. Blood of non-immunized and immunized sheep were analyzed in an autohematology analyzer (BC-5300 Vet; Mindray, China) and the percentage of lymphocytes **(B)** and neutrophils **(C)** based on the total white blood cells were analyzed at days 0, 3, 5, 7, 10 and 12 post-infection. Points indicate the individual value of each animal and lines represent the mean value of each group. No statistical differences were found by two-way ANOVA (*post hoc* Tukey test for multiple comparisons).

We also measured changes in rectal temperatures and hematological parameters in immunized and non-immunized sheep. After challenge with BTV-8, either the non-immunized or the MVA-VP2-NS1-2A-NS2-Nt developed a steep rise in their rectal temperatures between 4 and 6 d.p.i. (**Figure 2A**). No statistical differences regarding rectal temperatures were observed between both groups. However, whereas control animals maintained elevated rectal temperatures between days 6 and 9 post-infection, animals immunized with MVA-VP2-NS1-2A-NS2-Nt displayed a noticeable dumping of this temperature increase (**Figure 2A**). Similarly, we did not find statistical differences regarding percentages of neutrophils and lymphocytes in blood of immunized and non-immunized animals. Nonetheless, some control animals (sheep 2 and 4) showed elevated levels of neutrophils and reduced levels of lymphocytes at 3, 5 and 7 d.p.i. (**Figures 2B, C**). On the contrary, all animals immunized with MVA-VP2-NS1-2A-NS2-Nt presented steady levels of neutrophils and lymphocytes throughout the experiment (**Figures 2B, C**), which indicated the absence of neutrophilia and lymphopenia in these immunized sheep.

**Figure 3:**
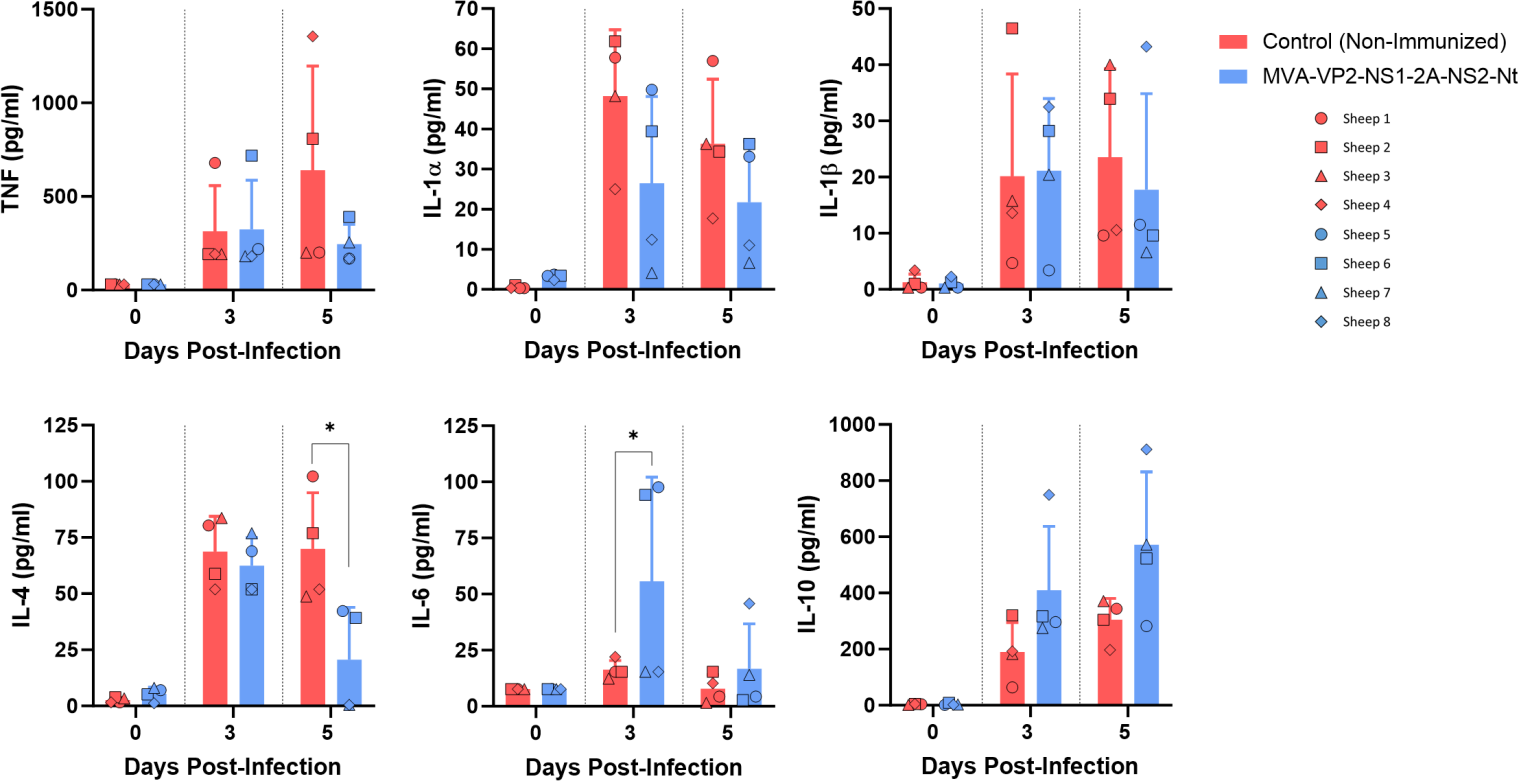
Levels of cytokines in serum samples from non-immunized and immunized sheep after challenge with BTV-8. Sheep sera were collected at 3 and 5 d.p.i. and analyzed in Luminex immunoas-says. Points represent individual values for each sheep, bars represent mean values of each group, and error bars represent SD. Asterisks denote significant differences between immunized and non-immunized control. * P value <  0.05, using two-way ANOVA (*post hoc* Tukey test for multiple comparisons).

In addition, the dynamics of circulating pro and anti-inflammatory cytokines in serum at 3 and 5 days after challenge was studied. Extensive changes in the profile of several of these cytokines were detected between vaccinated and non-vaccinated sheep at day 5 post-infection (**Figure 3**). At this day, increased levels of IL-1α, IL-1β, IL-4 and TNF were observed in non-vaccinated sheep compared with vaccinated animals. For IL-4, these differences were significant at 5 d.p.i. The excessive pro-inflammatory cytokine production seen in non-immunized sheep could contribute to the severity of disease. In contrast, immunized sheep downregulated these cytokine levels leading to a more balanced immune response. Interestingly, IL-10 was upregulated in vaccinated sheep after challenge at 3 and 5 d.p.i. (**Figure 3**). This cytokine play important roles as a regulatory cytokine and, in the context of an infectious disease, as IL-10 helps to prevent tissue damage caused by the immune response (29). In the case of IL-6, a cytokine with a pleiotropic effect, its levels were significant higher in vaccinated sheep just at 3 d.p.i., going down again at 5 d.p.i.

Overall, all these data reflect that immunization with MVA-VP2-NS1-2A-NS2-Nt can induce a high degree of heterologous protection against BTV, impeding viral replication to a high extent. Besides, this vaccine candidate may attenuate the severity of disease although it should be further confirmed in future histopathological studies.

### 3.2 Immunogenicity of the MVA-VP2-NS1-2A-NS2-Nt vaccine candidate in sheep

Thereafter, we aimed to characterize the immunogenicity of the MVA-VP2-NS1-2A-NS2-Nt in sheep. To that end, PBMCs and sera from immunized and non-immunized animals were harvested at 3 weeks post-boost.

**Figure 4:**
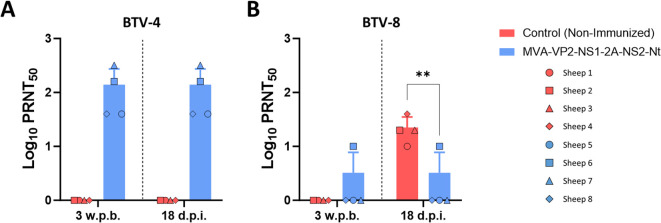
Neutralizing antibodies titers against **(A)** BTV-4M and **(B)** BTV-8 in immunized and non-immunized sheep by PRNT50 assay. NAbs titers were measured in sera collected at 3 w.p.b. and 18 d.p.i. after challenge with BTV-8. Points represent individual values for each sheep, bars represent the mean values of each group, points indicate the mean value of each group and error bars represent SD. **P value < 0.005, using two-way ANOVA (*post hoc* Tukey test for multiple comparisons).

First, we analyzed the induction of a neutralizing humoral immune response against BTV. As we observed in a previous study (23), immunization with MVA-VP2-NS1-2A-NS2-Nt induces a homologous neutralizing response against BTV-4 after two doses of the vaccine candidate (**Figure 4A**). We did not detect virus-neutralizing activity against the heterologous virus challenge BTV-8 (**Figure 4B**), which reflects that virus NAbs do not mediate protection against BTV-8 in immunized sheep. In contrast with the non-immunized control group, challenge with BTV-8 did not translate into a boost of NAbs levels against BTV-8 in the MVA-VP2-NS1-2A-NS2-Nt immunized animals (**Figure 4B**), which may be related to the attenuation of viral replication induced by the vaccine.

**Figure 5:**
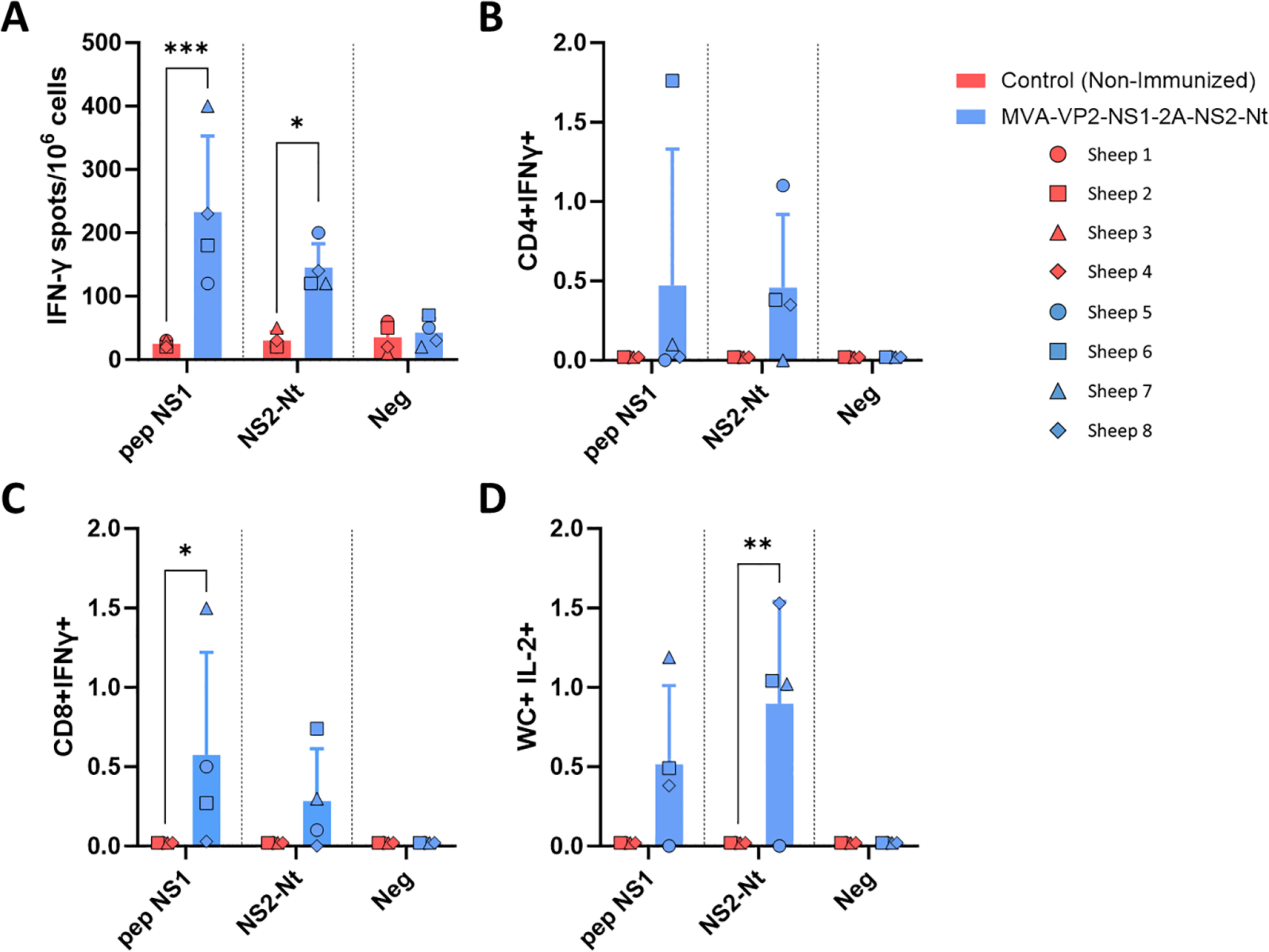
Cellular immune responses against BTV in MVA-VP2-NS1-2A-NS2-Nt immunized sheep. **(A)** ELISpot assay measuring IFN-γ-secreting cells after isolation of PBMCs of immunized and non-immunized sheep and stimulation with a pool of peptides of NS1, a recombinant protein NS2-Nt and an irrelevant peptide (neg). **(B–D)** Flow cytometry analysis. Percentage of CD4+IFN-γ+ **(B)**, CD8+ IFN-γ+ **(C)** and WC+IL-2+ **(D)** cells after stimulation of sheep PBMCs with a pool of peptides of NS1, a recombinant protein NS2-Nt and an irrelevant peptide. Points represent individual values for each sheep, bars represent mean values of each group and error bars represent the SD. *P value < 0.05, P value < 0.002, *P value < 0.0002 using two-way ANOVA (*post hoc* Tukey test for multiple comparisons). Neg: negative, irrelevant peptide from Gn RVFV.

In order to determine the ability of the proteins NS1 and NS2-Nt expressed by the MVA-VP2-NS1-2A-NS2-Nt to elicit specific T-cell protective immune responses in immunized sheep, we performed ELISpot and intracellular cytokine staining assays. At 3 weeks post-boost (w.p.b.), PBMCs from immunized and non-immunized sheep were restimulated for 18 h with a pool of NS1-specific T-cell peptides (**Table 1**), with baculovirus-expressed recombinant protein NS2-Nt, or with an irrelevant (off target) peptide from RVFV. Analysis of sheep PBMCs responses by IFN-γ ELISpot assay showed the induction of statistically significant BTV NS1 and NS2-Nt specific cellular immune responses in MVA-VP2-NS1-2A-NS2-Nt vaccinated animals (**Figure 5A**). By ICS, we observed that restimulation of sheep PBMCs with the NS1 peptide pool augmented CD4+IFN-γ+ T cell levels and significantly increased (p < 0.05) the expression of IFN-γ by CD8+ T cells from MVA-VP2-NS1-2A-NS2-Nt vaccinated sheep compared to controls (**Figures 5B, C**). After restimulation with protein NS2-Nt, we also observed increased levels of CD4+ and CD8+ T cells secreting IFN-γ compared to the non-immunized control group (**Figures 5B, C**). We also measured the levels of γδ T cells after restimulation of sheep PBMCs. γδ T-cells are a subset of non-conventional T cells highly present in ruminants and important in pathogen-infected cell clearance due to their potent cytotoxic and cytolytic functions as well as their capacity to modulate the adaptive immune response (30). Previous works have pointed out that WC1+ γδ T cells immunomodulates the adaptive immune response to a T-helper 1 (Th1)-like immune response (31). Considering that IL-2 is a strong immunoregulatory Th1 cytokine (32, 33), we gated these cells by expression of WC1 and IL-2 expression to identify γδ T cells that could potentially immunomodulate this kind of immune response. Restimulation of sheep PBMCs with the pool of NS1 peptides increased levels of WC1+IL-2+ T cells (**Figure 5D**). Similarly, a significant increase of γδ T-cells expressing IL-2 occurred after restimulation with protein NS2-Nt of PBMCs from MVA-VP2-NS1-2A-NS2-Nt vaccinated sheep (**Figure 5D**).

**Figure 6:**
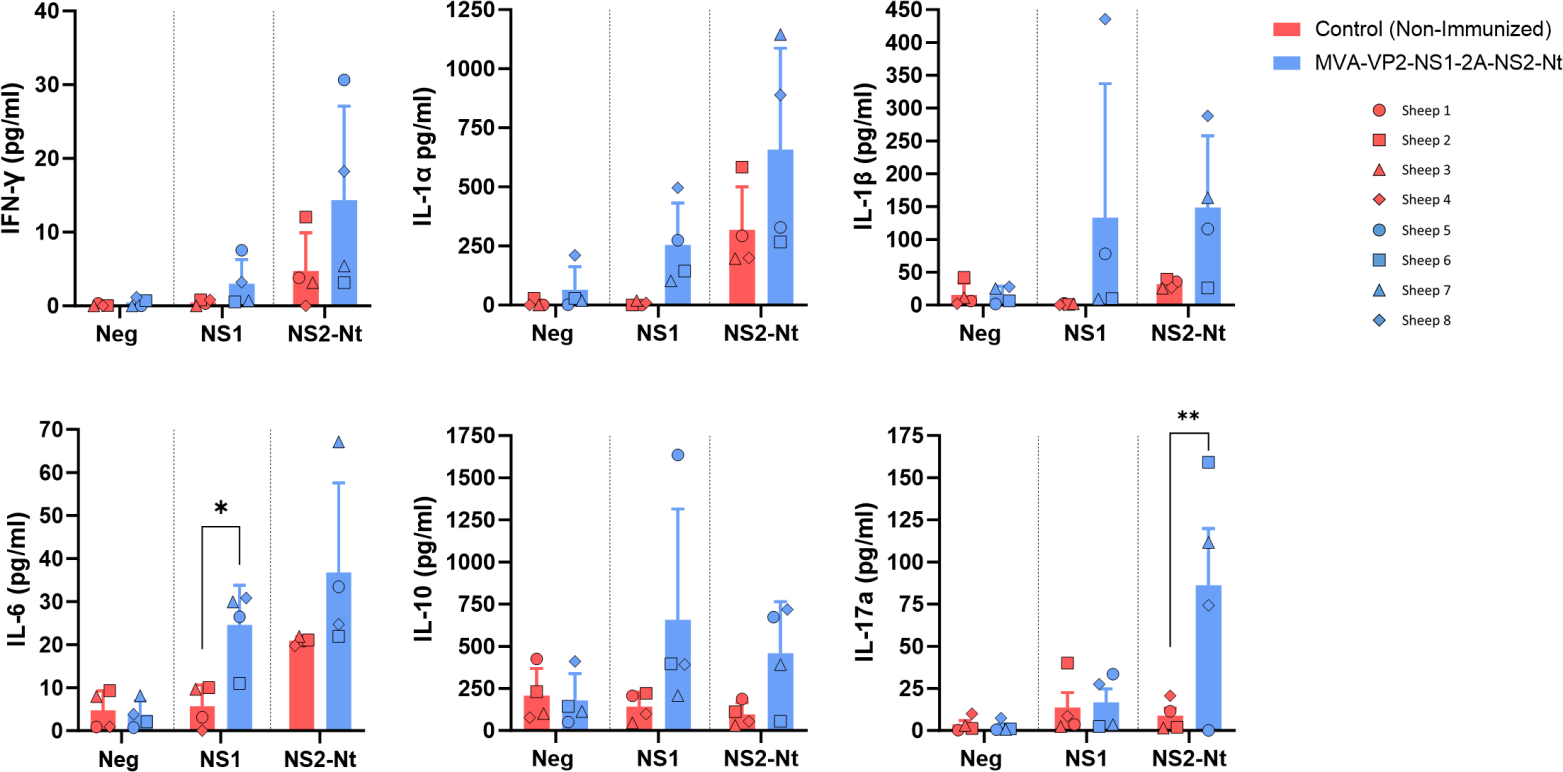
Secretion of cytokines after restimulation of PBMCs with NS1 peptides, NS2-Nt recombinant protein or an irrelevant antigen (Gn RVFV). Level of proteins (pg/ml) in cell supernatants at 18 h post incubation was analyzed by Luminex Multiplex assays. Points represent individual values for each sheep, bars represent mean values of each group and error bars represent the SD. *P value <  0.05, using two-way ANOVA (*post hoc* Tukey test for multiple comparisons) ** P value < 0.005.

On the other hand, PBMCs from sheep before challenge with BTV-8 were also used to study levels of cytokines released upon antigenic stimulation (NS1 or NS2-Nt) by luminex assay. Cells from vaccinated animals showed an increase in the secretion of several cytokines compared to controls. There was a specific induction of IFN-γ, IL-1α, IL-1β, IL-6, IL-10 and IL-17a (**Figure 6**) in response to NS1 peptides or NS2-Nt protein. These findings, together with the activation of lymphocytes previously seen in cytometry experiments, suggest that the vaccine successfully primes PBMCs for an enhanced immune response, which could be critical for effective protection against the targeted pathogen.

Overall, these results indicate that NS1 and NS2-Nt antigens, highly conserved among serotypes, expressed by the MVA-VP2-NS1-2A-NS2-Nt vaccine can induce strong T-cell responses in natural hosts of BTV, which eventually highlights the importance of the non-neutralizing adaptive immune response to provide protection against BTV.

## 4 Discussion

Inactivated and live-attenuated vaccines are the unique available prophylactic measures for effective control of BTV (8, 34). However, important drawbacks related to low immunogenicity and safety concerns restrict their use and raise an important need for recombinant vaccine development (8, 35). Moreover, vaccination against BTV implies to face two important aspects, e.g., the need for broad protective responses against the wide range of BTV serotypes and the implementation of a DIVA strategy. A high diversity of recombinant candidate vaccines have been developed for BTV (8, 20, 35, 36). However, the induction of broad protective responses elicited by these DIVA vaccines mostly rely on formulation of a set of components specific of each targeted serotype (37–40), which may impact vaccine affordability.

Recently, we have developed a recombinant MVA viral vector that co-expresses three BTV antigens, including the homologous NAbs-inducer protein VP2 and the highly conserved nonstructural proteins NS1 and NS2-Nt, responsible of the induction of potent T-cell responses (23). Previously, we observed that sheep immunization with MVA-VP2-NS1-2A-NS2-Nt induces a full degree of protection against a homologous serotype of BTV. It is important to note that we hypothesized that this high degree of protection was mainly mediated by the VP2-specific NAbs, without diminishing the role of the other two BTV antigens (23). The inclusion of NS1 and NS2-Nt, highly conserved proteins among serotypes, pursued both the reinforcement of the adaptive immune response induced by the vaccine and the elicitation of broad protective responses against multiple BTV serotypes. Now, we evaluated the capacity of this vaccine candidate to provide cross-protective responses against BTV. As we observed, immunization with MVA-VP2-NS1-2A-NS2-Nt partially protected against the heterologous BTV-8. It is understandable that, due to genomic diversity and the non-induction of NAbs specific of serotype 8, this vaccine candidate did not provide a level of protection against BTV-8 as high as it did against the homologous serotype, although it was still noteworthy, causing a significant reduction in the viremia in the immunized sheep. It is worth note that, alike NAbs-dependent protection, protection against BTV mediated by a T-cell response is usually related with detectable viral replication and mild disease (22, 41). Nonetheless, we observed that viral replication was significantly reduced after the challenge with BTV-8 in immunized animals. Besides, MVA-VP2-NS1-2A-NS2-Nt immunization seems to curb the pathology induced by BTV-8 in inoculated sheep, as reflected by suppression of IL-4 expression, a cytokine that may lead to a exacerbated inflammation and deleterious effects on virus clearance (42), as well as increased expression levels of IL-10, described as a central mediator of immune balance, helping to control excessive inflammation, preventing tissue damage, and limiting immune-related pathology (29, 43). The early and temporary up-regulation of IL-6 in vaccinated group at 5 d.p.i., could be related with its role in immune regulation. This cytokine has a pleiotropic effect and influences the differentiation and function of T helper (Th) cell subsets, regulating adaptive immune responses during infections (44). Therefore, the MVA-VP2-NS1-2A-NS2-Nt vaccine candidate is also efficacious against disease progression after infection with heterologous serotypes.

Importantly, we hypothesized that this vaccine candidate can block virus transmission to the insect vector as seen by the absence of viraemia or RNAemia in sheep after challenge with the homologous BTV-4M (23). In this sense, vaccination with MVA-VP2-NS1-2A-NS2-Nt also induced a significant impairment of viral replication after challenge with the heterologous BTV-8, which could also lead to impairment of the BTV transmission cycle. In this sense, *Culicoides* infection rates are low and dose-dependent (45–47), with an estimated 50% midge alimentary infective dose (MAID50) that far exceeds the highest titer of infectious virus observed in systemic blood of the MVA-VP2-NS1-2A-NS2-Nt immunized sheep. Besides, BTV virus titers in skin periphery seem to be much lower compared to those found in systemic blood after the beginning of infection (47). Thus, this recombinant vaccine candidate is potentially efficacious on blocking onward transmission to insect vector of both homologous and heterologous BTV serotypes.

Previous vaccination studies showed that heterologous combination of ChAdOx1 and MVA co-expressing proteins NS1 and NS2-Nt induces partial protection in sheep against a virulent challenge with BTV-4M (17). Despite that clinical disease is mitigated, viral replication still occurs at high level at early stages of infection followed by a faster viral clearance induced by the vaccine. In a following study, immunization with an MVA co-expressing proteins VP7, NS1 and NS2-Nt also led to similar results on protection against the same virulent strain (23). Now, we observe that protection mediated by nonstructural proteins NS1 and NS2-Nt delivered by the MVA-VP2-NS1-2A-NS2-Nt was significantly better against BTV-8 compared to those previous candidates. Considering the superior immunogenicity of heterologous prime-boost immunization strategies (48) or the potentially added immune response against protein VP7 (41, 49, 50), our results may be questionable. However, differences on virulence between strains and serotypes is a common feature of orbiviruses (2, 51–53). Therefore, the differential virulence between BTV-4M and BTV-8 could explain the greater protective response against BTV-8 induced by the non-structural proteins NS1 and NS2-Nt delivered by the MVA-VP2-NS1-2A-NS2-Nt. Nevertheless, the strain of BTV-8 (BEL/2006) used in these experiments was described as virulent in sheep during a major epidemic of BT in North-West Europe in 2006 (54, 55). However, absence or mild BT-related disease can occur in experimental infection with BTV isolates observed as virulent in the field (56). Other virulence determinants related to the mammalian host breed/age, immunological status and/or a possible attenuation due to virus isolation in cell culture may be also a reasonable explanation (2, 57). In this sense, animal breed used in the present study may be different from local breeds in North-West Europe.

Cytotoxic T lymphocytes play a key role in protection against BTV infection (22). Non-structural proteins of BTV are the main source of T cell epitopes (58). For NS1, lymphoproliferative responses in cattle already indicated the potential of this BTV-antigen to stimulate cytotoxic T responses (59), which coincide with data regarding the immunogenicity of the MVA-VP2-NS1-2A-NS2-Nt in sheep. In mice and cattle, the capacity of protein NS2 to induce T-cell mediated responses is also known (59–61). Recently, we determined that the N-terminal half of NS2, NS2-Nt, contains the majority of T-cell epitopes of NS2, demonstrating that the response induced by NS2-Nt is equal to that induced by NS2 in mice (18). Nonetheless, it was unknown whether the immunogenicity of NS2-Nt in mice corresponded to a similar immune response in natural hosts of BTV. Here, we observed that the NS2-Nt protein promotes BTV-specific CD4+, CD8+ and γδ T cell responses after vaccination of sheep with MVA-VP2-NS1-2A-NS2-Nt. Therefore, the formulation of the N-terminal half of NS2 does not impact immunogenicity of this antigen in BTV-natural hosts.

Cytokine secretion following antigen stimulation is key for evaluating the efficacy of vaccines. Upon stimulation of immunized sheep PBMCs with NS1 peptides or recombinant NS2-Nt antigen, there was an evident production of a variety of cytokines that play crucial roles in immune responses, such as IFN-γ, IL-1α, IL-1β, IL-6, IL-10 and IL-17a. These cytokines play an essential role in mounting an effective immune response against different viruses, as described previously in other studies (62, 63). In particular, there was a statistically significant up-regulation of IL-6 and IL-17a. Among IL-6 functions, this cytokine have a role in the differentiation of CD8^+^ T cells into cytotoxic T lymphocytes (64). In the case of IL-17a, this is produced by Th17 CD4+ T cells that play a role in vaccine-induced memory immune responses (65). Overall, we have confirmed that the highly conserved NS1 and NS2-Nt antigens co-delivered by the same MVA viral vector are able to trigger multifunctional multiserotype responses against BTV in a natural host of the disease, which is a major aspect in recombinant vaccine research against BTV.

γδ T cells are non-conventional T cells that comprise up to 60% of circulating lymphocytes in young cattle and sheep, and constitute a comparable proportion of PBMCs to those of CD8+ T cells and CD4+ T cells in adult animals (66–70). This cell subset actively participates in protective immunity in mammals against a variety of pathogens (67). Here, sheep developed a significant gamma-delta response specific to NS1 and NS2-Nt after vaccination with MVA-VP2-NS1-2A-NS2-Nt. γδ T cells also act as a bridging between innate and adaptive immunity, resulting in a T h1-biased response (68). In this sense, γδ T cells induced by the MVA-VP2-NS1-2A-NS2-Nt vaccine also expressed IL-2, a cytokine that promotes a variety of effector cytotoxic CD8+ (Th1) T cell responses (71). In most ruminants, γδ T cells are prominent in the epithelial tissues including the skin, where WC1+ γδ T cells are predominant (69, 72). Despite that we observed a BTV-specific WC1+ γδ T cells response in PBMCs, it could replicate in other tissues where WC1+ γδ T cells are abundant, such as the dermis (73), which may reinforce the first immunological barrier at the site of infection. γδ T cells are recruited to inflamed areas of the skin (69), which could be a consequence of midge feeding. Importantly, considering that BTV can infect this T cell subset, which might increase viral replication at biting sites (74), further research is warrant to elucidate how vaccination influences the role of γδ T cells during BTV infection.

In summary, here we confirmed the multiserotype protective capacity of the MVA-VP2-NS1-2A-NS2-Nt DIVA vaccine candidate in sheep. Importantly, this work suggests the role of the T-cell responses specific of the nonstructural proteins NS1 and NS2 on mediating broad protection against BTV in the natural host.

## Data Availability

The original contributions presented in the study are included in the article/**Supplementary Material**. Further inquiries can be directed to the corresponding author.

## Supplementary material

Supplementary Figure 1 Gating strategy for flow cytometry analysis. Representative dot plots gating lymphocytes **(A)**, single **(B)** and live cells **(C)**. Frequencies of T cell subsets including CD4+ **(D)**, CD8+ **(E)** and γδ + cells **(F)** are shown.

## References

[1] Carpenter S Wilson A Mellor PS. Culicoides and the emergence of bluetongue virus in northern Europe. Trends Microbiol. (2009) 17:172–8. doi: 10.1016/j.tim.2009.01.001 19299131

[2] Caporale M Di Gialleonorado L Janowicz A Wilkie G Shaw A Savini G. Virus and host factors affecting the clinical outcome of bluetongue virus infection. J Virol. (2014) 88:10399–411. doi: 10.1128/JVI.01641-14 PMC4178883 24991012

[3] Maan S Maan NS Samuel AR Rao S Attoui H Mertens PPC. Analysis and phylogenetic comparisons of full-length VP2 genes of the 24 bluetongue virus serotypes. J Gen Virol. (2007) 88:621–30. doi: 10.1099/vir.0.82456-0 17251581

[4] Kundlacz C Caignard G Sailleau C Viarouge C Postic L Vitour D. Bluetongue virus in France: an illustration of the European and Mediterranean context since the 2000s. Viruses. (2019) 11. doi: 10.3390/v11070672 PMC6669443 31340459

[5] Holwerda M Santman-Berends IMGA Harders F Engelsma M Vloet RPM Dijkstra E. Emergence of bluetongue virus serotype 3 in the Netherlands in September 2023. Emerg Infect Dis. (2023) 30(8):1552–61. doi: 10.1101/2023.09.29.560138 PMC11286052 38941965

[6] Rushton J Lyons N. Economic impact of Bluetongue: a review of the effects on production. Vet Ital. (2015) 51:401–6. doi: 10.12834/VetIt.646.3183.1 26741252

[7] Gethmann J Probst C Conraths FJ. Economic impact of a bluetongue serotype 8 epidemic in Germany. Front Vet Sci. (2020) 7:65. doi: 10.3389/fvets.2020.00065 32118078 PMC7034324

[8] van Rijn PA. Prospects of next-generation vaccines for bluetongue. Front Vet Sci. (2019) 6:407. doi: 10.3389/fvets.2019.00407 31824966 PMC6881303

[9] Alonso C Utrilla-Trigo S Calvo-Pinilla E Jiménez-Cabello L Ortego J Nogales A. Inhibition of orbivirus replication by aurintricarboxylic acid. Int J Mol Sci. (2020) 21. doi: 10.3390/ijms21197294 PMC7582255 33023235

[10] Matthijnssens J Attoui H Bányai K Brussaard CPD Danthi P Del Vas M. ICTV virus taxonomy profile: sedoreoviridae 2022. J Gen Virol. (2022) 103. doi: 10.1099/jgv.0.001782 PMC12643109 36215107

[11] Patel A Roy P. The molecular biology of Bluetongue virus replication. Virus Res. (2014) 182:5–20. doi: 10.1016/j.virusres.2013.12.017 24370866 PMC7162684

[12] Grimes JM Burroughs JN Gouet P Diprose JM Malby R Ziéntara S. The atomic structure of the bluetongue virus core. Nature. (1998) 395:470–8. doi: 10.1038/26694 9774103

[13] Ratinier M Caporale M Golder M Franzoni G Allan K Nunes SF. Identification and characterization of a novel non-structural protein of bluetongue virus. PloS Pathog. (2011) 7:e1002477. doi: 10.1371/journal.ppat.1002477 22241985 PMC3248566

[14] Mohd Jaafar F Monsion B Mertens PPC Attoui H. Identification of orbivirus non-structural protein 5 (NS5), its role and interaction with RNA/DNA in infected cells. Int J Mol Sci. (2023) 24:6845. doi: 10.3390/ijms24076845 37047816 PMC10095184

[15] Schwartz-Cornil I Mertens PPC Contreras V Hemati B Pascale F Bréard E. Bluetongue virus: virology, pathogenesis and immunity. Vet Res. (2008) 39:46. doi: 10.1051/vetres:2008023 18495078

[16] Fay PC Mohd Jaafar F Batten C Attoui H Saunders K Lomonossoff GP. Serological cross-reactions between expressed VP2 proteins from different bluetongue virus serotypes. Viruses. (2021) 13:1455. doi: 10.3390/v13081455 34452321 PMC8402635

[17] Utrilla-Trigo S Jiménez-Cabello L Alonso-Ravelo R Calvo-Pinilla E Marín-López A Moreno S. Heterologous combination of chAdOx1 and MVA vectors expressing protein NS1 as vaccination strategy to induce durable and cross-protective CD8+ T cell immunity to bluetongue virus. Vaccines. (2020) 8:346. doi: 10.3390/vaccines8030346 32610561 PMC7564706

[18] Utrilla-Trigo S Jiménez-Cabello L Calvo-Pinilla E Marín-López A Lorenzo G Sánchez-Cordón P. The Combined Expression of the Non-structural Protein NS1 and the N-Terminal Half of NS2 (NS21-180) by ChAdOx1 and MVA Confers Protection against Clinical Disease in Sheep upon Bluetongue Virus Challenge. J Virol. (2021) 96(3):e01614–21. doi: 10.1128/JVI.01614-21 34787454 PMC8826911

[19] Calvo-Pinilla E Marín-López A Moreno S Lorenzo G Utrilla-Trigo S Jiménez-Cabello L. A protective bivalent vaccine against Rift Valley fever and bluetongue. NPJ Vaccines. (2020) 5:1–12. doi: 10.1038/s41541-020-00218-y 32793399 PMC7393076

[20] Jiménez-Cabello L Utrilla-Trigo S Calvo-Pinilla E Moreno S Nogales A Ortego J. Viral vector vaccines against bluetongue virus. Microorganisms. (2020) 9. doi: 10.3390/microorganisms9010042 PMC7823852 33375723

[21] Andrew M Whiteley P Janardhana V Lobato Z Gould A Coupar B. Antigen specificity of the ovine cytotoxic T lymphocyte response to bluetongue virus. Vet Immunol Immunopathol. (1995) 47:311–22. doi: 10.1016/0165-2427(94)05410-T 8571549

[22] Jeggo MH Wardley RC Brownlie J. Importance of ovine cytotoxic T cells in protection against bluetongue virus infection. Prog Clin Biol Res. (1985) 178:477–87. 2989889

[23] Jiménez-Cabello L Utrilla-Trigo S Calvo-Pinilla E Lorenzo G Illescas-Amo M Benavides J. Co-expression of VP2, NS1 and NS2-Nt proteins by an MVA viral vector induces complete protection against bluetongue virus. Front Immunol. (2024) 15:1440407. doi: 10.3389/fimmu.2024.1440407 39072326 PMC11272488

[24] Nomikou K Hughes J Wash R Kellam P Breard E Zientara S. Widespread reassortment shapes the evolution and epidemiology of bluetongue virus following European invasion. PloS Pathog. (2015) 11:e1005056. doi: 10.1371/journal.ppat.1005056 26252219 PMC4529188

[25] Marín-López A Bermúdez R Calvo-Pinilla E Moreno S Brun A Ortego J. Pathological characterization of IFNAR(-/-) mice infected with bluetongue virus serotype 4. Int J Biol Sci. (2016) 12:1448–60. doi: 10.7150/ijbs.14967 PMC5166487 27994510

[26] Calvo-Pinilla E Rodríguez-Calvo T Sevilla N Ortego J. Heterologous prime boost vaccination with DNA and recombinant modified vaccinia virus Ankara protects IFNAR(–/–) mice against lethal bluetongue infection. Vaccine. (2009) 28:437–45. doi: 10.1016/j.vaccine.2009.10.027 19857449

[27] Marín-López A Utrilla-Trigo S Jiménez-Cabello L Ortego J. Recombinant Modified Vaccinia Virus AnkaraModified vaccinia virus Ankara (MVA) Development to Express VP2, NS1, and VP7 Proteins of Bluetongue VirusBluetongue virus (BTV). In: Brun A, editor. Vaccine Technologies for Veterinary Viral Diseases: Methods and Protocols. Methods in Molecular Biology. Springer US, New York, NY (2022). p. 177–93. doi: 10.1007/978-1-0716-2168-4_10 35118622

[28] Toussaint JF Sailleau C Breard E Zientara S De Clercq K. Bluetongue virus detection by two real-time RT-qPCRs targeting two different genomic segments. J Virological Methods. (2007) 140:115–23. doi: 10.1016/j.jviromet.2006.11.007 17196266

[29] Carlini V Noonan DM Abdalalem E Goletti D Sansone C Calabrone L. The multifaceted nature of IL-10: regulation, role in immunological homeostasis and its relevance to cancer, COVID-19 and post-COVID conditions. Front Immunol. (2023) 14:1161067. doi: 10.3389/fimmu.2023.1161067 37359549 PMC10287165

[30] Chan KF Duarte JDG Ostrouska S Behren A. γδ T cells in the tumor microenvironment-interactions with other immune cells. Front Immunol. (2022) 13:894315. doi: 10.3389/fimmu.2022.894315 35880177 PMC9307934

[31] Kennedy HE Welsh MD Bryson DG Cassidy JP Forster FI Howard CJ. Modulation of immune responses to Mycobacterium bovis in cattle depleted of WC1(+) gamma delta T cells. Infect Immun. (2002) 70:1488–500. doi: 10.1128/IAI.70.3.1488-1500.2002 PMC127737 11854237

[32] Yue M Deng X Zhai X Xu K Kong J Zhang J. Th1 and Th2 cytokine profiles induced by hepatitis C virus F protein in peripheral blood mononuclear cells from chronic hepatitis C patients. Immunol Lett. (2013) 152:89–95. doi: 10.1016/j.imlet.2013.05.002 23680070

[33] Saxena R Kaur J. Th1/Th2 cytokines and their genotypes as predictors of hepatitis B virus related hepatocellular carcinoma. World J Hepatol. (2015) 7:1572–80. doi: 10.4254/wjh.v7.i11.1572 PMC4462695 26085916

[34] Feenstra F van Rijn PA. Current and next-generation bluetongue vaccines: Requirements, strategies, and prospects for different field situations. Crit Rev Microbiol. (2017) 43:142–55. doi: 10.1080/1040841X.2016.1186005 27800699

[35] Jiménez-Cabello L Utrilla-Trigo S Barreiro-Piñeiro N Pose-Boirazian T Martínez-Costas J Marín-López A. Nanoparticle- and microparticle-based vaccines against orbiviruses of veterinary importance. Vaccines (Basel). (2022) 10:1124. doi: 10.3390/vaccines10071124 35891288 PMC9319458

[36] Calvo-Pinilla E Castillo-Olivares J Jabbar T Ortego J de la Poza F Marín-López A. Recombinant vaccines against bluetongue virus. Virus Res. (2014) 182:78–86. doi: 10.1016/j.virusres.2013.11.013 24287057

[37] Roy P Bishop DH LeBlois H Erasmus BJ. Long-lasting protection of sheep against bluetongue challenge after vaccination with virus-like particles: evidence for homologous and partial heterologous protection. Vaccine. (1994) 12:805–11. doi: 10.1016/0264-410x(94)90289-5 7975859

[38] Pérez de Diego AC Athmaram TN Stewart M Rodríguez-Sánchez B Sánchez-Vizcaíno JM Noad R. Characterization of protection afforded by a bivalent virus-like particle vaccine against bluetongue virus serotypes 1 and 4 in sheep. PloS One. (2011) 6:e26666. doi: 10.1371/journal.pone.0026666 22046324 PMC3202233

[39] Celma CCP Boyce M van Rijn PA Eschbaumer M Wernike K Hoffmann B. Rapid generation of replication-deficient monovalent and multivalent vaccines for bluetongue virus: protection against virulent virus challenge in cattle and sheep. J Virol. (2013) 87:9856–64. doi: 10.1128/JVI.01514-13 PMC3754119 23824810

[40] van Rijn PA Maris-Veldhuis MA Spedicato M Savini G van Gennip RGP. Pentavalent disabled infectious single animal (DISA)/DIVA vaccine provides protection in sheep and cattle against different serotypes of bluetongue virus. Vaccines (Basel). (2021) 9:1150. doi: 10.3390/vaccines9101150 34696258 PMC8537505

[41] Wade-Evans AM Romero CH Mellor P Takamatsu H Anderson J Thevasagayam J. Expression of the major core structural protein (VP7) of bluetongue virus, by a recombinant capripox virus, provides partial protection of sheep against a virulent heterotypic bluetongue virus challenge. Virology. (1996) 220:227–31. doi: 10.1006/viro.1996.0306 8659119

[42] Sharma DP Ramsay AJ Maguire DJ Rolph MS Ramshaw IA. Interleukin-4 mediates down regulation of antiviral cytokine expression and cytotoxic T-lymphocyte responses and exacerbates vaccinia virus infection *in vivo* . J Virol. (1996) 70:7103–7. doi: 10.1128/JVI.70.10.7103-7107.1996 PMC190762 8794356

[43] Rojas JM Avia M Martín V Sevilla N. IL-10: A multifunctional cytokine in viral infections. J Immunol Res. (2017) 2017:6104054. doi: 10.1155/2017/6104054 28316998 PMC5337865

[44] Uciechowski P Dempke WCM. Interleukin-6: A masterplayer in the cytokine network. Oncology. (2020) 98:131–7. doi: 10.1159/000505099 31958792

[45] Veronesi E Darpel K Gubbins S Batten C Nomikou K Mertens P. Diversity of transmission outcomes following co-infection of sheep with strains of bluetongue virus serotype 1 and 8. Microorganisms. (2020) 8:851. doi: 10.3390/microorganisms8060851 32516979 PMC7356686

[46] van Gennip RGP Drolet BS Rozo Lopez P Roost AJC Boonstra J van Rijn PA. Vector competence is strongly affected by a small deletion or point mutations in bluetongue virus. Parasites Vectors. (2019) 12:470. doi: 10.1186/s13071-019-3722-2 31604476 PMC6790033

[47] Melzi E Caporale M Rocchi M Martín V Gamino V di Provvido A. Follicular dendritic cell disruption as a novel mechanism of virus-induced immunosuppression. Proc Natl Acad Sci U S A. (2016) 113:E6238–47. doi: 10.1073/pnas.1610012113 PMC5068271 27671646

[48] Palgen J-L Feraoun Y Dzangué-Tchoupou G Joly C Martinon F Le Grand R. Optimize prime/boost vaccine strategies: trained immunity as a new player in the game. Front Immunol. (2021) 12:612747. doi: 10.3389/fimmu.2021.612747 33763063 PMC7982481

[49] Rojas JM Barba-Moreno D Avia M Sevilla N Martín V. Vaccination with recombinant adenoviruses expressing the bluetongue virus subunits VP7 and VP2 provides protection against heterologous virus challenge. Front Vet Sci. (2021) 8:645561. doi: 10.3389/fvets.2021.645561 33778041 PMC7987666

[50] Martín V Pascual E Avia M Peña L Valcárcel F Sevilla N. Protective efficacy in sheep of adenovirus-vectored vaccines against bluetongue virus is associated with specific T cell responses. PloS One. (2015) 10. doi: 10.1371/journal.pone.0143273 PMC4664254 26619062

[51] Drolet BS Reister LM Rigg TD Nol P Podell BK Mecham JO. Experimental infection of white-tailed deer (Odocoileus virginianus) with Northern European bluetongue virus serotype 8. Vet Microbiol. (2013) 166:347–55. doi: 10.1016/j.vetmic.2013.05.027 23876932

[52] Jimenez-Cabello L Utrilla-Trigo S Benavides J Anguita J Calvo-Pinilla E Ortego J. IFNAR(-/-) mice constitutes a suitable animal model for epizootic hemorrhagic disease virus study and vaccine evaluation. Int J Biol Sci. (2024) 20(8):3076–93. doi: 10.7150/ijbs.95275 PMC11186350 38904031

[53] Janowicz A Caporale M Shaw A Gulletta S Di Gialleonardo L Ratinier M. Multiple genome segments determine virulence of bluetongue virus serotype 8. J Virol. (2015) 89:5238–49. doi: 10.1128/JVI.00395-15 PMC4442542 25822026

[54] Elbers ARW van der Spek AN van Rijn PA. Epidemiologic characteristics of bluetongue virus serotype 8 laboratory-confirmed outbreaks in The Netherlands in 2007 and a comparison with the situation in 2006. Preventive Vet Med. (2009) 92:1–8. doi: 10.1016/j.prevetmed.2009.08.007 19740560

[55] Elbers ARW Backx A Meroc E Gerbier G Staubach C Hendrickx G. Field observations during the bluetongue serotype 8 epidemic in 2006. I. Detection of first outbreaks and clinical signs in sheep and cattle in Belgium, France and the Netherlands. Prev Vet Med. (2008) 87:21–30. doi: 10.1016/j.prevetmed.2008.06.004 18620767

[56] Schulz C Bréard E Sailleau C Jenckel M Viarouge C Vitour D. Bluetongue virus serotype 27: detection and characterization of two novel variants in Corsica, France. J Gen Virol. (2016) 97:2073–83. doi: 10.1099/jgv.0.000557 27435041

[57] Herder V Caporale M MacLean OA Pintus D Huang X Nomikou K. Correlates of disease severity in bluetongue as a model of acute arbovirus infection. PloS Pathog. (2024) 20:e1012466. doi: 10.1371/journal.ppat.1012466 39150989 PMC11357116

[58] Jones LD Chuma T Hails R Williams T Roy P. The non-structural proteins of bluetongue virus are a dominant source of cytotoxic T cell peptide determinants. J Gen Virol. (1996) 77:997–1003. doi: 10.1099/0022-1317-77-5-997 8609498

[59] Anderson J Hägglund S Bréard E Comtet L Lövgren Bengtsson K Pringle J. Evaluation of the immunogenicity of an experimental subunit vaccine that allows differentiation between infected and vaccinated animals against bluetongue virus serotype 8 in cattle. Clin Vaccine Immunol. (2013) 20:1115–22. doi: 10.1128/CVI.00229-13 PMC3754508 23720365

[60] Anderson J Bréard E Lövgren Bengtsson K Grönvik K-O Zientara S Valarcher J-F. Purification, stability, and immunogenicity analyses of five bluetongue virus proteins for use in development of a subunit vaccine that allows differentiation of infected from vaccinated animals. Clin Vaccine Immunol. (2014) 21:443–52. doi: 10.1128/CVI.00776-13 PMC3957662 24451327

[61] Anderson J Hägglund S Bréard E Riou M Zohari S Comtet L. Strong protection induced by an experimental DIVA subunit vaccine against bluetongue virus serotype 8 in cattle. Vaccine. (2014) 32:6614–21. doi: 10.1016/j.vaccine.2014.09.066 25312275

[62] Kostinov MP Akhmatova NK Khromova EA Kostinova AM. Cytokine profile in human peripheral blood mononuclear leukocytes exposed to immunoadjuvant and adjuvant-free vaccines against influenza. Front Immunol. (2020) 11:1351. doi: 10.3389/fimmu.2020.01351 32695114 PMC7339108

[63] Al-Qahtani AA Alhamlan FS Al-Qahtani AA. Pro-inflammatory and anti-inflammatory interleukins in infectious diseases: A comprehensive review. Trop Med Infect Dis. (2024) 9:13. doi: 10.3390/tropicalmed9010013 38251210 PMC10818686

[64] Tanaka T Narazaki M Kishimoto T. IL-6 in inflammation, immunity, and disease. Cold Spring Harb Perspect Biol. (2014) 6:a016295. doi: 10.1101/cshperspect.a016295 25190079 PMC4176007

[65] McGeachy MJ. Th17 memory cells: live long and proliferate. J Leukoc Biol. (2013) 94:921–6. doi: 10.1189/jlb.0313113 24006508

[66] Mackay CR Maddox JF Brandon MR. Three distinct subpopulations of sheep T lymphocytes. Eur J Immunol. (1986) 16:19–25. doi: 10.1002/eji.1830160105 3081353

[67] Mackay CR Hein WR. A large proportion of bovine T cells express the gamma delta T cell receptor and show a distinct tissue distribution and surface phenotype. Int Immunol. (1989) 1:540–5. doi: 10.1093/intimm/1.5.540 2535142

[68] Baldwin CL Yirsaw A Gillespie A Le Page L Zhang F Damani-Yokota P. γδ T cells in livestock: Responses to pathogens and vaccine potential. Transbound Emerg Dis. (2020) 67 Suppl 2:119–28. doi: 10.1111/tbed.13328 31515956

[69] Baldwin CL Sathiyaseelan T Rocchi M McKeever D. Rapid changes occur in the percentage of circulating bovine WC1(+)gamma delta Th1 cells. Res Vet Sci. (2000) 69:175–80. doi: 10.1053/rvsc.2000.0410 11020371

[70] Baldwin CL Damani-Yokota P Yirsaw A Loonie K Teixeira AF Gillespie A. Special features of γδ T cells in ruminants. Mol Immunol. (2021) 134:161–9. doi: 10.1016/j.molimm.2021.02.028 33774521

[71] Smith HE Jacobs RM Smith C. Flow cytometric analysis of ovine peripheral blood lymphocytes. Can J Vet Res. (1994) 58:152–5. PMC1263683 8004542

[72] Kalia V Sarkar S. Regulation of effector and memory CD8 T cell differentiation by IL-2-A balancing act. Front Immunol. (2018) 9:2987. doi: 10.3389/fimmu.2018.02987 30619342 PMC6306427

[73] Geherin SA Lee MH Wilson RP Debes GF. Ovine skin-recirculating γδ T cells express IFN-γ and IL-17 and exit tissue independently of CCR7. Vet Immunol Immunopathol. (2013) 155:87–97. doi: 10.1016/j.vetimm.2013.06.008 23838472 PMC3982400

[74] Hein WR Dudler L. TCR gamma delta+ cells are prominent in normal bovine skin and express a diverse repertoire of antigen receptors. Immunology. (1997) 91:58–64. doi: 10.1046/j.1365-2567.1997.00224.x 9203966 PMC1364035

