# Supplementary figures and images for: The MVA-VP2-NS1-2A-NS2-Nt vaccine candidate provides heterologous protection in sheep against bluetongue virus
Source: Front Immunol. 2025 May 5;16:1566225. doi: 10.3389/fimmu.2025.1566225 (PMC12086148; doi:10.3389/fimmu.2025.1566225)

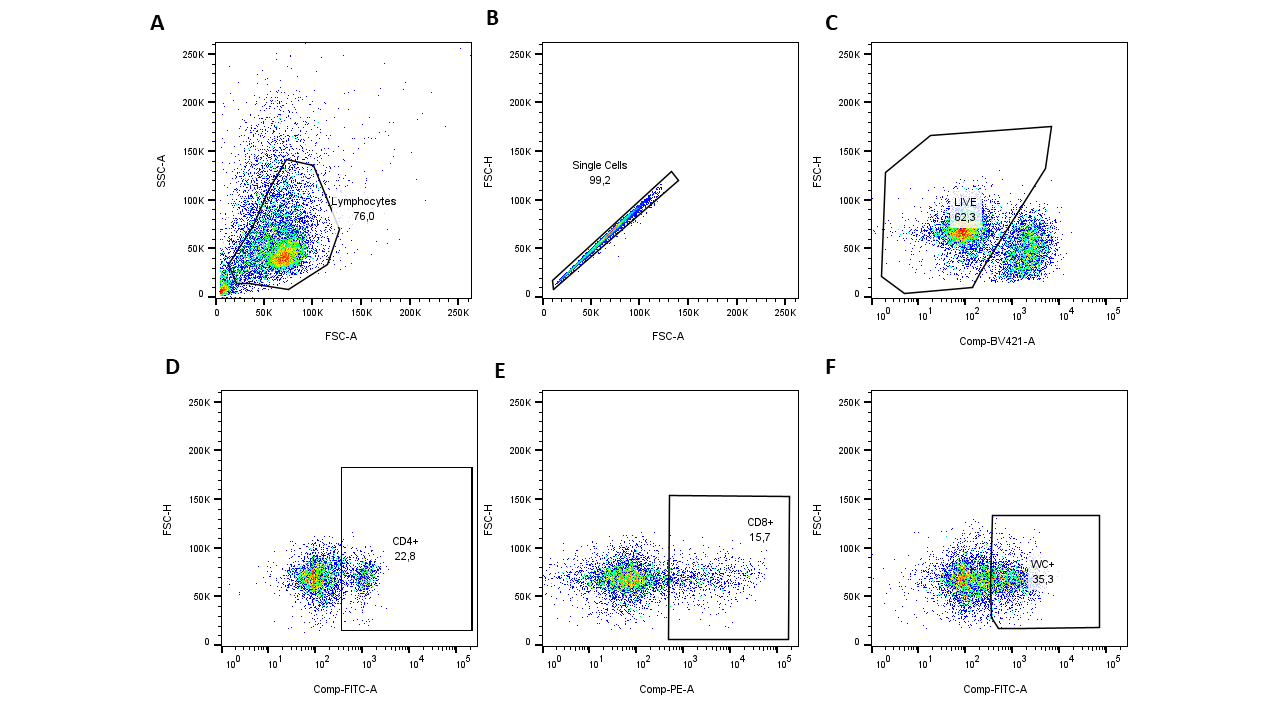

Supplement: Supplementary Figure 1 — Gating strategy for flow cytometry analysis. Representative dot plots gating lymphocytes (A), single (B) and live cells (C). Frequencies of T cell subsets including CD4+ (D), CD8+ (E) and γδ + cells (F) are shown. [file Image1.tif]
